# Binding Direction-Based Two-Dimensional Flattened Contact Area Computing Algorithm for Protein–Protein Interactions

**DOI:** 10.3390/molecules22101722

**Published:** 2017-10-13

**Authors:** Beom Sik Kang, GaneshKumar Pugalendhi, Ku-Jin Kim

**Affiliations:** 1 School of Life Sciences and Biotechnology, Kyungpook National University, 80 Daehakro, Bukgu, Daegu 41566, Korea; bskang2@knu.ac.kr; 2 Department of Information Technology, Anna University Regional Campus, Coimbatore 641046, Tamil Nadu, India; ganesh23508@gmail.com; 3 School of Computer Science and Engineering, Kyungpook National University, 80 Daehakro, Bukgu, Daegu 41566, Korea

**Keywords:** protein-protein interface, contact area, binding direction, protein docking

## Abstract

Interactions between protein molecules are essential for the assembly, function, and regulation of proteins. The contact region between two protein molecules in a protein complex is usually complementary in shape for both molecules and the area of the contact region can be used to estimate the binding strength between two molecules. Although the area is a value calculated from the three-dimensional surface, it cannot represent the three-dimensional shape of the surface. Therefore, we propose an original concept of two-dimensional contact area which provides further information such as the ruggedness of the contact region. We present a novel algorithm for calculating the binding direction between two molecules in a protein complex, and then suggest a method to compute the two-dimensional flattened area of the contact region between two molecules based on the binding direction.

## 1. Introduction

Proteins are involved in almost all biological processes, such as gene expression, cell proliferation and cell signaling. Most proteins work as a multiprotein complex or together with partner proteins and rarely function alone. Interactions between protein molecules are essential for the function of the proteins, including multimeric assembly like cytoskeleton and viral capsid. Proteins form a transient complex like signal transducing proteins or a permanent complex as a certain multi-subunit enzyme. In such protein–protein interactions, the contact region between the two protein molecules is referred to as the binding surface of the proteins. An analysis of the protein binding surfaces could provide an understanding of functions and action mechanisms of the proteins in the complex, while also offering information for the dynamics of the complexes and development of drugs that can interfere with these interactions [1,2,3,4,5,6,7,8].

Since analyzing the features of the contact regions between two protein molecules is very important for various usages, many research attempts have already been made [9,10,11,12,13,14,15,16]. They provide information such as the area of binding surface, the binding strength, residues involved in the binding, and the types of their interaction.

In a natural state, a protein assembly can exist under certain conditions and yet dissociate under other conditions. It depends on the binding strength of the proteins and possible modifications of the binding surface. Protein–protein interactions can be stabilized mostly by ionic interactions, hydrogen-bond, and hydrophobic interaction. The binding strength may be higher with a greater binding surface area as more atoms participate in the interaction between the two molecules. In particular, a hydrophobic interaction is the most important factor for protein binding in an aquatic solution as it removes the water molecules surrounding the hydrophobic regions of the proteins. Thus, when two proteins are combined, the water molecules surrounding the proteins are reduced according to the binding area, which means the binding area can represent the strength of the protein–protein interaction. Therefore, many programs have been developed to calculate the binding area for solvation. However, the solvation area is a two-dimensional value, and cannot present the three-dimensional shape of the binding surface whether it is flat or has ruggedness.

Lee and Richards [17] introduced the concept of solvent accessible surface area (SASA) in 1971, where solvent accessible surface is the center trajectory of the solvent which rolls and contacts all over the molecule without penetration. When the molecule is represented by a set of van der Waals spheres, the solvent accessible surface corresponds to the offset surface of the spheres with the radius of the solvent.

Wodak and Janin [18] suggested a method to compute SASA by using an analytical approximation. They derived the analytic equation to compute the solvation surface area for two atoms by computing the surface area of two intersecting spheres $S_{1}$ and $S_{2}$, with the radii $r_{i}+r_{w}$, *i* = 1,2, where $r_{i}$ is the van der Waals radius of the corresponding atom and $r_{w}$ is the radius of the water molecule. For more than two intersecting spheres, it is hard to analytically compute the surface area, because of the buried surfaces. Therefore they approximated the solvation surface area of atoms by computing the probability for a point on the surface of the sphere to be outside of all intersecting spheres based on the analytic equation.

Richmond [19] proposed an analytical way to compute SASA. They derived an analytic formula to compute the spherical surface area which is bounded by piecewise regular curves by using the concepts from differential geometry. Then, SASA is computed by evaluating the formula. They also presented an analytical formula for computing the enclosed volume by the solvent accessible surface.

For the binding specificity in protein–protein interactions, two major factors are complementary shape and charges of the binding area between two proteins. To evaluate or predict the binding interface of one protein to its real or potential partners, the ruggedness, which is a numerical value describing the degree of prominence and depression on the binding surface, is an important factor. It puts weight, on shape rather than charge, for evaluating binding specificity.

When we imagine an island, the two-dimensional area in a map helps to estimate the size of the island whether it has a big mountain or not. If we apply this concept to a binding interface of a protein, two-dimensional flatted area for the interface can present a size for the interface other than the solvation area. Additionally, by dividing the solvation area value with flatted area of the binding surface, we can obtain numerical value describing the degree of prominence or depression on the binding surface. Thus, it is necessary to define a sea level for the proteins in a complex representing the flattened binding interface.

According to the ‘collision theory’ explanation of chemical reaction rates, the impact between reactants in the right direction with sufficient energy leads to a successful reaction [20]. For the binding reaction of two protein molecules, they must approach each other according to the direction of their binding interfaces in order to make a complex. Thus, considering the protein contact region in terms of the appropriate angle is also important for a protein–protein interaction. Therefore, identifying the binding direction of proteins in a complex can reveal the appropriate orientation required to create a complex between the partner proteins. This binding direction can then be used to find sectional planes, which are perpendicular to the direction. One of the sectional planes in the complex can be considered as the sea level for the binding area of a protein like an island, and the two-dimensional flattened binding area can be calculated.

This paper presents a novel approach for estimating the interaction between two proteins in a protein complex from coordinate files containing three-dimensional protein structures, which are deposited in Protein Data Bank. First, the binding direction d of two protein molecules is computed, and the contact area is then calculated with respect to d. A comparison of the sectional area and contact area of the proteins in the protein complex is also used to estimate the degree of prominence and depression on the binding surface. Finally, an example of further analysis for the binding interface is suggested using a series of the sectional area like contour lines in a map.

This paper is organized as follows. Section 2 discusses related work. Section 3 explains the proposed method for deciding the binding direction, while Section 4 explains the proposed method for computing the sectional area of the contact region. Experimental results are presented in Section 5, and some final conclusions are given in Section 6.

## 2. Related Work

The PSIMAP algorithm uses the Euclidean distance between two protein structures to check their interaction [9,10,21]. Thus, the contact region between two protein structures is determined based on the five-five rule, which assumes that two protein molecules only interact with each other if there are at least five residue pairs within a 5 $\overset{˚}{A}$. The Sampled Atom Contact PSIMAP and Full Atom Contact PSIMAP are two variants of the basic PSIMAP algorithm.

Dafas et al. [22] improved on the basic PSIMAP with the bounding box contact PSIMAP algorithm. In this algorithm, each protein structure is bounded with a bounding shape (bounding box or convex hull), thereby restricting the search space for interacting atoms to the intersecting area of the two bounding shapes. The application of parallel processing when computing the interacting atoms also dramatically improved the computation time. PSIbase [10] is also a database used to construct an interactome map for two protein structures based on the PSIMAP algorithm.

Nye et al. [23] proposed a method to predict the domain–domain contact of protein super families based on the statistical significance of interacting protein pairs measured using a *p*-value. However, this approach is not consistently effective as the training genomic data does not contain clear information. Hence, Nye et al. [11] improved the prediction strength by defining the contact area of a superfamily using the number of atoms within a 5 $\overset{˚}{A}$ from another molecule, which is not found in experimental data. Meanwhile, Steinkellner et al. [13] detected the contact patches from crystals, and analyzed them according to the RNA, DNA, or ligand contacts. To determine the interactions between protein structures, they used the solvent-excluded surface (SES) for each protein structure, measured the distance between point pairs from two solvent-excluded surfaces, and then determined the contact patches according to the distance cutoff.

Arab et al. [14] also presented a method for computing the potential energy of protein contacts based on the pairwise residue contact area. They defined the contact area as the spherical surface of the residue atoms contacting with the other molecule. They located probes at sample points on the surface of the atoms, and considered the number of points that intersected with the other molecule as the contact area.

PISA [16,24] is a software tool that computes the contact area of a protein complex using a finite element analysis as the average difference between the surface area of each molecular component and the total accessible surface area. Plus, the graph theoretical approach [12] was proposed to discover the assemblies in a crystal using an assembly enumeration algorithm, where the chemical stability of an assembly is determined using the consequences of entropy in the case of molecules bound to proteins.

In [15], the issue of docking failure due to the falsification of protein-protein interactions in crystals is addressed. PISA is used to select the dimeric structure in the PDB (Protein Data Bank). A fast Fourier transform is used to find the relative position between two protein molecules, and secondary structure matching is used to align the residues.

CAPRI [25] is a protein–protein structure interaction prediction experiment and provides a forum for analyzing protein-docking methods in the case of blind prediction. A user can obtain the atomic coordinates of two proteins that make organically significant interactions, model a complex of the proteins, and submit their results for valuation on the CAPRI Web site. Since macromolecular interactions are a central theme in functional genomics, this promotes the development of prediction methods for the association of two proteins based on their three-dimensional structure.

Gu et al. [26] presented a method for protein–protein docking based on shape complementarity. They characterized the local shape of each protein surface by using the surface-histogram. By measuring the distance between two surface-histograms, they found the possible contact region between two proteins.

In this paper, we use ePISA to select a protein complex in the PDB for the experiments, since it provides information for the interfaces and assemblies of all components in an asymmetric unit and symmetry-related molecules in the given crystal structure.

## 3. Computation of Binding Direction

To the best of our knowledge, there have not been previous researches to compute the binding direction between two molecules in a protein complex. As a novel attempt, we propose a method for computing the binding direction based on the visibility concept from the computational geometry area. When a protein complex is composed of three or more molecules, the proposed algorithm can be directly applied to compute the binding direction between each pair of molecules. The protein complex is represented by a PDB file (http://www.pdb.org) that includes a snapshot location of every atom’s center in the complex.

From a geometric perspective, a protein molecule is generally represented as a set of spheres, where each atom in the protein molecule corresponds to a sphere with a Van der Waals radius. Let $M_{A}$ denote the set of spheres corresponding to one molecule in the given complex, while $M_{B}$ denotes the set of spheres corresponding to the other molecule in the complex.

**Figure 1:**
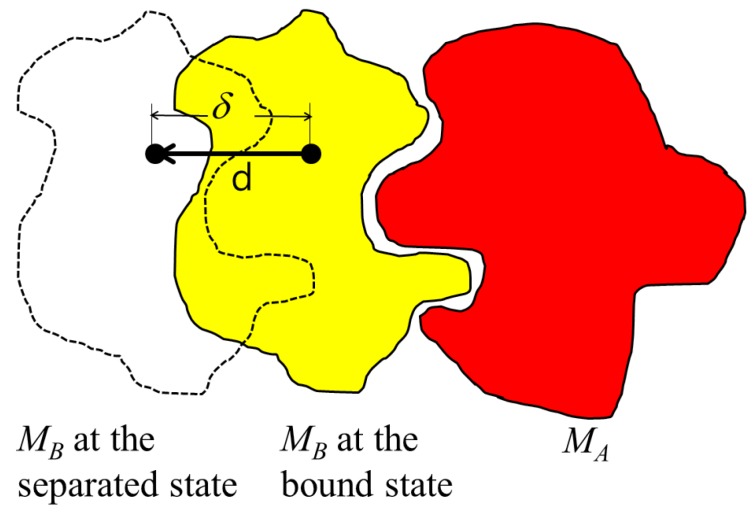
Binding direction versus separating direction.

While the PDB file provides the location and orientation of rigid bodies $M_{A}$ and $M_{B}$ that are already bound, the binding direction is unknown. Thus, the binding direction of $M_{B}$ with respect to $M_{A}$ is computed using the separating direction based on the location of the atoms in $M_{A}$ and $M_{B}$ in the protein complex. The separating direction is the direction in which $M_{B}$ moves away from $M_{A}$ without any collisions. As such, the separating direction can be considered as opposite of the binding direction. In Figure 1, the separating direction is denoted as d, where the binding direction is −d.

The separating direction is computed based on determining the moving direction of $M_{B}$ when it does not intersect with $M_{A}$ while moving within distance δ. When the restriction of the maximum moving distance is given as δ, we select the atoms that never collide with the partner molecule within δ. For each atom in $M_{B}$, the closest atoms in $M_{A}$ are computed, along with the minimum distance between them. If the minimum distance is less than δ, there is a possibility that $M_{A}$ and $M_{B}$ may collide when $M_{B}$ is moving. Otherwise, $M_{A}$ and $M_{B}$ will not collide when $M_{B}$ is moving, as the distance between them is longer than the maximum moving distance of $M_{B}$. Thus, the selected set of atoms is $A\in M_{A}$ and $B\in M_{B}$ where the minimum distance from the atom in *A* to the atom in *B* is less than or equal to δ.

**Figure 2:**
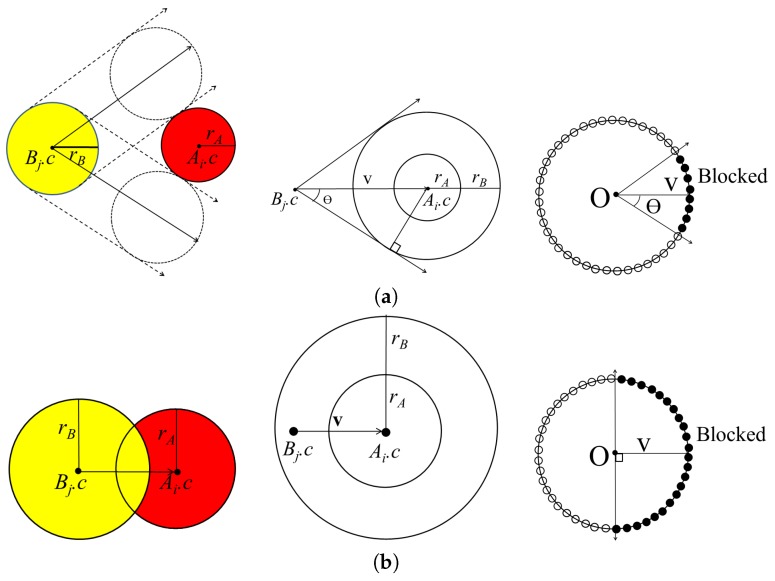
Illustration of atoms from *A* and *B*, and construction of binding cones (**a**) case when $A_{i}$ and $B_{j}$ do not intersect and (**b**) case when $A_{i}$ and $B_{j}$ intersect with each other.

First, we compute the moving directions of all the atoms in *B* that are blocked by atoms in *A*. By computing the union set of the blocked directions for each atom, we can then compute the blocked direction for the molecule $M_{B}$. Let $A_{i}$ and $B_{j}$ denote an atom in *A* and *B* with centers $A_{i}.c$ and $B_{j}.c$, respectively. When $B_{j}$ moves along a linear trajectory, it is blocked by $A_{i}$ in a certain direction. The union of the directions blocked by $A_{i}$ is represented by a cone centered at $B_{j}.c$. The cone K(v,θ) is constructed, where *K* is the vertex at the origin, the axis $v=A_{i}.c-B_{j}.c$, and the half-angle θ:

$$\theta =sin{}^{-1}\left(\frac{R}{∥v∥}\right),$$

if $B_{j}.c$ is not contained in the sphere with center $A_{i}.c$ and radius $A_{i}.r+B_{j}.r$. Figure 2a shows the cone constructed for $A_{i}$ and $B_{j}$.

In some cases, atoms from different molecules intersect with each other when there is a strong bond between them. For this case, atoms $A_{i}$ and $B_{j}$ can be separated in the opposite direction in the half-space constructed by a plane with reference point $A_{i}.c$ and normal vector $A_{i}.c-B_{j}.c$. Figure 2b shows the cone constructed for $A_{i}$ and $B_{j}$ with axis $A_{i}.c-B_{j}.c$ and half-angle π/2.

**Figure 3:**
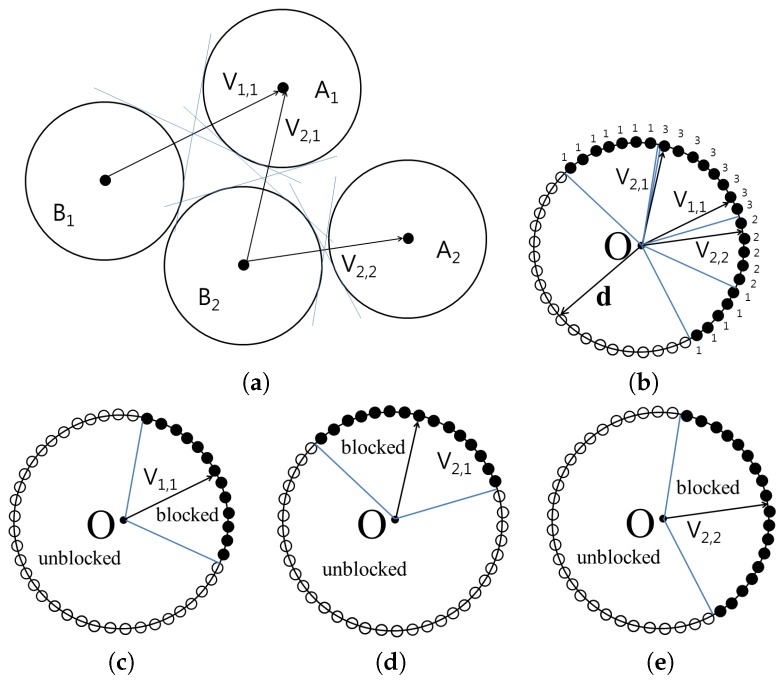
Example of computing blocked directions. (**a**) the relative positions of $A_{1}$, $A_{2}$, $B_{1}$ and $B_{2}$; (**b**) the cone consructed for three pairs ($A_{1}$, $B_{1}$), ($A_{1}$, $B_{2}$) and ($A_{2}$, $B_{2}$); (**c**) the cone for $A_{1}$ and $B_{1}$; (**d**) the cone for $A_{1}$ and $B_{2}$; and (**e**) the cone for $A_{2}$ and $B_{2}$.

The Figure 3 shows the cone constructed for the set $A=\{A_{1},A_{2}\}$ and $B=\{B_{1},B_{2}\}$. The vertices of the cones containing blocked directions are then translated to the origin in order to compute the union of the cones. From the origin, all possible directions are represented as surface points on a unit sphere. For each point p on the unit sphere, the number of cones that encompass p is counted and stored as p.count. If p.count is 0, $M_{A}$ and $M_{B}$ can be separated by translating $M_{B}$ using vector (0,0,0)−p with distance δ without collision. However, if p.count is *n*, n>0, $M_{A}$ and $M_{B}$ can be separated by translating $M_{B}$ using vector (0,0,0)−p with distance δ, and there will be *n* collisions.

When $P^{\prime }=\left\{p\;|\;p.count=0\right\}$, every separating direction can be found using the points in $P^{\prime }$. $P^{\prime }$ can be composed of one or more segments. Each sample point on the unit sphere keeps information on the neighbor points. To group the connected points in $P^{\prime }$ as one segment, we used the function **GroupingPoints** that is based on the flood-filling method [27]. Flood-filling method is used in computer graphics applications to recursively fill the area surrounded by a closed boundary starting from a seed point in the area. From an arbitrary point $p\in P^{\prime }$, we apply the function **GroupingPoints** to group the connected points from **p** as $G_{1}$. After assigning group number $G_{1}$ to every point connected from **p**, another seed point from $P^{\prime }$ whose group is not decided yet is chosen. Then, we can apply the function **GroupingPoints** from it to detect the points which will be in group $G_{2}$. The process is repeated until every point in $P^{\prime }$ is assigned its group number.
**Function GroupingPoints** (**p**)/* **p**: a point in P’ */**Begin** **if** the group of **p** is not decided yet **then** **begin**  Set the group **p** as *G*_1_;  **for each p**_*i*_ ∈ *P*’, where **p**_*i*_ is a neighbor of **p**, **do**   **GroupingPoints** (**p**_*i*_); **end****End**

When p,q∈P are given, their groups are identical, if p and q can be connected by neighbors, and different if p and q are not connected. After deciding the groups for each point in $P^{\prime }$, the representative direction is determined for each group. In the algorithm **BindingDirection**, the separating direction d is computed as the average of the unblocked directions, and the binding direction −d is then returned.

**Function BindingDirection($M_{A}$, $M_{B}$, δ)**/* $M_{A}=\left\{A_{i}\;\right|\;A_{i}$ is a ball representing an atom, $0\leq i<n_{A}\}$ *//* $M_{B}=\left\{B_{j}\;\right|\;B_{j}$ is a ball representing an atom, $0\leq j<n_{B}\}$ *//* δ : maximum moving distance */**Begin** *A* = the atoms in $M_{A}$ whose minimum distance to $M_{B}$ is less than δ; *B* = the atoms in $M_{B}$ whose minimum distance from $M_{A}$ is less than δ; *P* = {sample points on the unit sphere}; Set p.count=0 for every p∈P; **for each**
$A_{i}\in A$
**do begin**  **for each**
$B_{j}\in B$
**do begin**    $v=B_{j}.c-A_{i}.c$;    **if** dist$(A_{i},B_{j})\leq 0$
**then** // Refer Figure 2a     Construct an infinite cone *K* with vertex (0,0,0),      axis *v*, and half-angle π/2;    **else if** dist$(A_{i},B_{j})\leq \delta$
**then** //Refer Figure 2b     Construct an infinite cone *K* with vertex (0,0,0), axis *v*,      and half-angle $\theta =sin^{-1}\left(\frac{A_{i}.r+B_{j}.r}{∥v∥}\right)$;    **for each**
p∈P
**do**     Increment p.count if p is inside *K*; // Refer Figure 3b  **end** **end** **d** = the average of the vectors p−(0,0,0), for p whose p.count is less than the given threshold; **return** −**d**;**End**

## 4. Computing Sectional Contact Area

**Function DistMap($M_{A}$, $M_{B}$)**/* $M_{A}$ and $M_{B}$ are transformed molecules */**Begin** Generate a view volume as the bounding box containing $M_{A}$ and $M_{B}$; NormFact := the size of the view volume along *z*-axis; Create two windows $W_{1}$ and $W_{2}$ with w×h size; // When the depth buffer is rendered, //   the depth values are automatically normalized to the range [0, 1] In $W_{1}$, render the depth buffer for $M_{A}$ with viewpoint (0, 0, −∞)  in orthographic view, then save the result to DA[w][h]; // Figure 5b In $W_{2}$, render the depth buffer for molecule $M_{B}$ with viewpoint (0, 0, +∞)  in orthographic view, then save the result to DB[w][h]; // Figure 5c **For each**
*i*, *j*
**do**  DB[i][j] = 1 - DB[i][j]; // Figure 5d **For each**
*i*, *j*
**do begin**  **if** DA[i][j] is 0 or 1 **then**   DA[i][j] = ∞;  **if** DB[i][j] is 0 or 1 **then**   DB[i][j] = ∞; **end** Create distMap[w][h]; **For each** i,j **do**  if (DA[i][j] != ∞ AND DB[i][j] != ∞)   distMap[i][j] = (DA[i][j] - DB[i][j])*NormFact; // Figure 5e  else distMap[i][j] = ∞; **return** distMap;**End**

**Figure 4:**
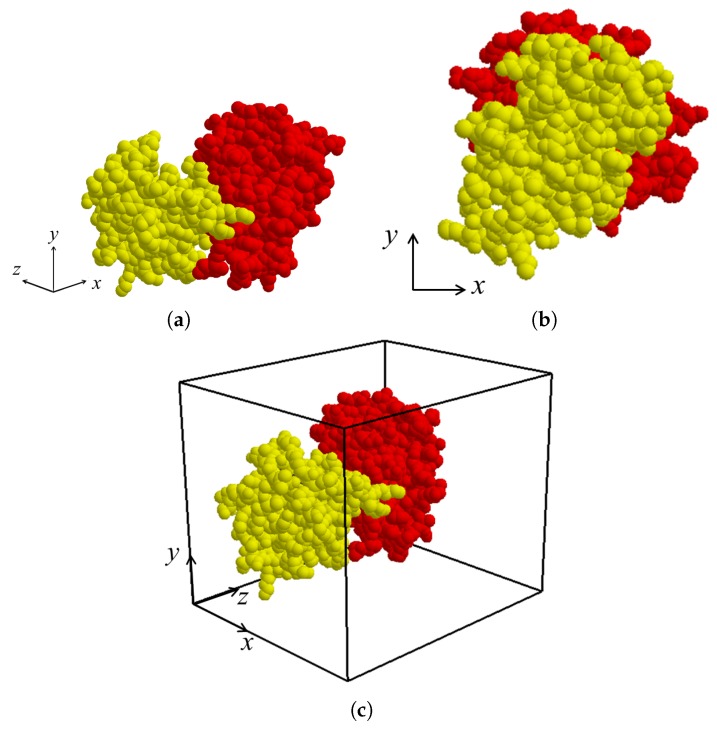
Result of updating orientation of protein complex (PDB id. 2HSN) according to binding direction: (**a**) molecules $M_{A}$ and $M_{B}$ in original position; (**b**) after rigid-body transformation of $M_{A}$ and $M_{B}$ to locate binding direction along *z*-axis; and (**c**) generated view volume for transformed $M_{A}$ and $M_{B}$.

After the binding direction for $M_{A}$ and $M_{B}$ in the protein complex is determined as −d, the binding direction is located along the *z*-axis by applying rigid-body rotation and translation for both molecules $M_{A}$ and $M_{B}$ if necessary (Figure 4a,b). The view volume for the protein complex can then be constructed as an axis-aligned bounding box containing both $M_{A}$ and $M_{B}$. (Figure 4c). Thereafter, the sectional contact area of $M_{A}$ and $M_{B}$ is computed using a depth buffer that renders the molecules within the view volume.

A depth buffer is used for hidden surface removal in the OpenGL graphics library (http://www.opengl.org). When three-dimensional objects are rendered using the OpenGL library, a depth buffer is used to store the minimum distance information of the rendered objects from the view plane. For example, when rendering a protein molecule using a depth buffer, each screen pixel should render a particular part of the molecule. When a pixel corresponds to several parts, the part closest to the view plane is rendered. Thus, a depth buffer keeps the minimum distance value from the view plane to the object for each pixel. The depth value is computed by selecting the closest part to the projection plane, and implemented using hardware, so the computation speed is very fast and the resolution easily adjusted by rendering the object larger or smaller.

**Figure 5:**
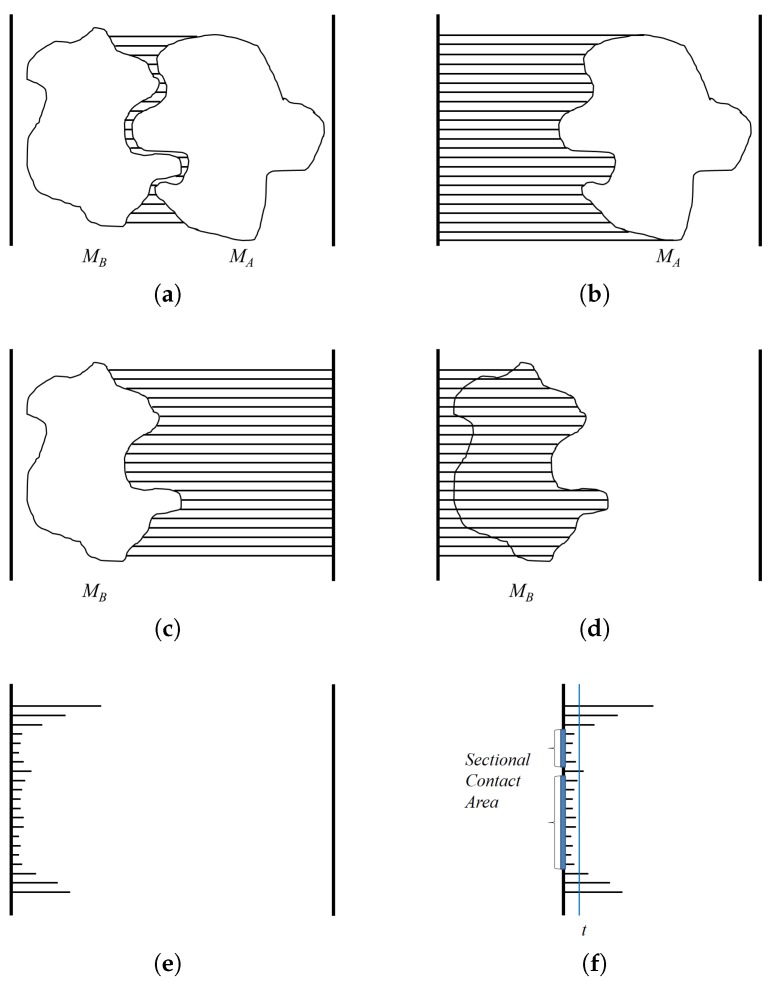
Computation of the distance and contact area between two molecules using depth buffer: (**a**) the distance between $M_{A}$ and $M_{B}$; (**b**) the depth values of $M_{A}$ from viewpoint (0,0,−∞); (**c**) the depth values of $M_{B}$ from viewpoint (0,0,∞); (**d**) the subtracted depth values of $M_{B}$; (**e**) the distance values between $M_{A}$ and $M_{B}$; and (**f**) the sectional contact area.

The goal of using a depth buffer is to compute the minimum distance between two molecules *A* and *B* along the binding direction (Refer Figure 5a). When a depth buffer is used, the resulting depth values are normalized between zero and one, where one corresponds to the view volume size along the *z*-axis. An orthogonal projection is also used so the given viewpoint actually works as a view direction. For the case of $M_{A}$, the depth values are computed for viewpoint (0,0,−∞) (Figure 5b). However, for the case of $M_{B}$, the depth values are computed for viewpoint (0,0,∞) (Figure 5c). After computing the depth values for viewpoint (0,0,∞), the distance to the binding surface of $M_{B}$ in the opposite direction can be computed by subtracting the depth values from one (Figure 5d). Meanwhile, subtracting the depth values of $M_{B}$ from $M_{A}$ reveals the distance between $M_{A}$ and $M_{B}$ (Figure 5e). The actual distance can be derived from the normalized distance by multiplying the view volume size along the *z*-axis. The algorithm **DistMap** shows the procedure for computing the distance between two molecules along the binding direction.

The depth values of interest are restricted to the contact region of $M_{A}$ and $M_{B}$. The algorithm **ContactArea** shows the procedure for computing the sectional area between $M_{A}$ and $M_{B}$. Along the binding direction, when the threshold for the contact region is given as *t*, we consider the pixels in the algorithm **DistMap** that show the distance as smaller than or equal to *t* as in the contact region. The pixels with a distance value that satisfies this condition are counted, and the area of the contact region is then computed by multiplying the unit area for each pixel. The unit area for a pixel *u* is computed using the ratio between the window size of the screen coordinate and the width and height of the three-dimensional view volume (Figure 5f).

**Figure 6:**
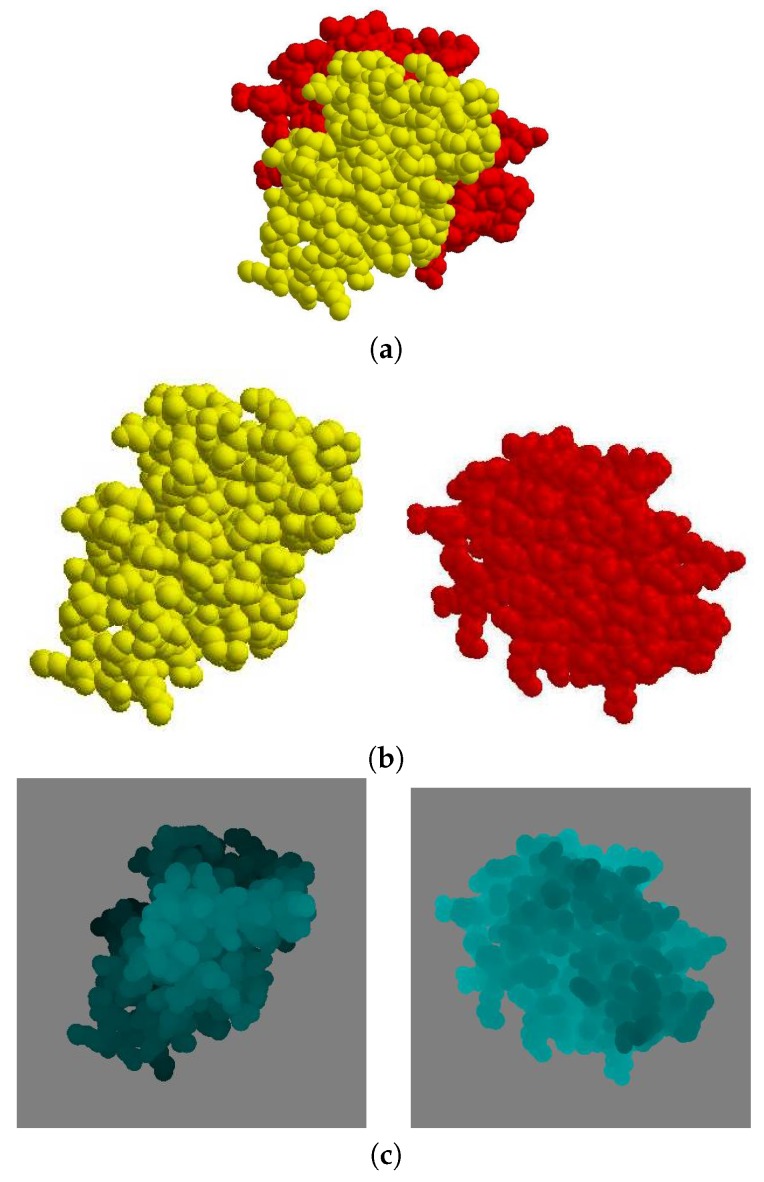
Depth buffer-rendered images of $M_{A}$ and $M_{B}$ in molecule (PDB id. 2HSN): (**a**) $M_{A}$ and $M_{B}$; (**b**) $M_{A}$ and $M_{B}$ are shown separately; and (**c**) the depth buffer images of $M_{A}$ and $M_{B}$.

Given a protein complex in a crystal that has a binding direction coincident with the −z axis (Figure 6a), the separate images of each molecule $M_{A}$ and $M_{B}$ are shown in (Figure 6b). The depth buffer images of $M_{A}$ and $M_{B}$ are then shown in (Figure 6c), where the gray color pixels are the background and the other pixels are the molecule. When the depth value is higher, the corresponding pixel in the image is brighter.

**Figure 7:**
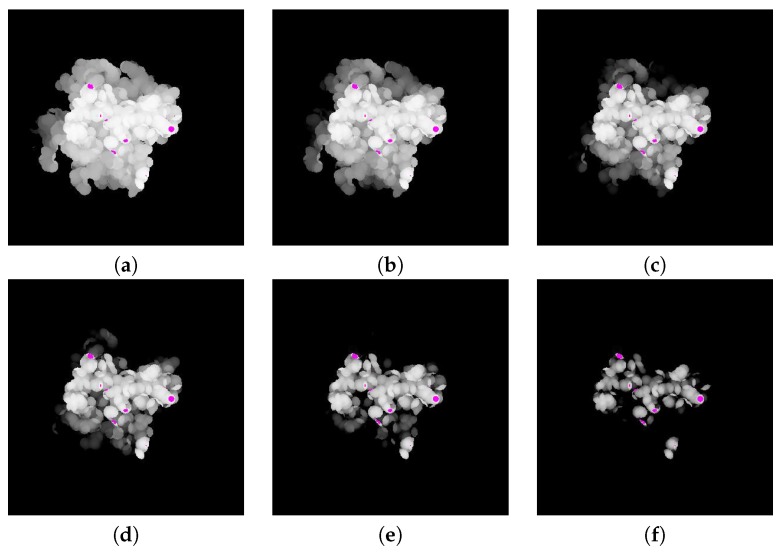
Difference images of depth values: (**a**) original difference image and (**b**–**f**) quantized difference images using thresholds of 30, 20, 15, 10, and five, respectively.

The difference between the depth values of $M_{A}$ and $M_{B}$ is computed by subtracting the depth value of $M_{B}$ from $M_{A}$. The resulting difference image is shown in Figure 7.

**Function ContactArea($M_{A}$, $M_{B},\;t,\;u$)**/* $M_{A}$, $M_{B}$ : molecules *//* *t*: threshold for meaningful distance *//* *u*: unit area for one pixel */**Begin** Area = 0; **For each** distMap[i][j] **begin**  **if** distMap[i][j] ≤*t*
**then**   Area=Area + distMap[i][j]; **End** **Return**
Area*u;**End**

## 5. Experimental Results

In the previous section, the binding direction was determined based on the best approach for two proteins to bind with each other. The perpendicular plane to the binding direction was used to view the binding interface and describe the binding surface in a two-dimensional way. Based on the perpendicular plane, a two-dimensional sectional binding area of the proteins was calculated for the viewpoint of the binding direction. This sectional area can also be used to analyze the protein–protein interaction, where the shape of the binding surface can be very diverse.

Similar to two molecules in an enzyme-substrate interaction or ligand-receptor binding, two proteins in a complex have a binding specificity and this specificity comes from the reciprocal shape of their binding surfaces, like a lock-and-key. Thus, the prominence and depression patterns found on the surface of one protein are the opposite patterns found on its binding partner. Of course, the specificity also includes additional components, such as the pairing of opposite electro potentials, where a negatively charged region is matched with a positively charged region, while neutral regions provide hydrophobic interactions. In some protein–protein interactions, the binding region can be quite flat. In this case, the specificity is mainly derived from the potentials of the surface or the proteins could be promiscuous. The flatness of the binding surfaces can be estimated by comparing the two-dimensional sectional area with the three-dimensional surface area of the binding interface, thereby revealing the degree of convexity or concaveness of the binding surfaces.

To obtain the coordinates of protein–protein complexes, 32 crystal structures were randomly selected from a protein data bank. The binding direction of the proteins in the 32 complexes were then determined and the binding interfaces were analyzed (Table 1). The sectional area of the proteins in each complex was calculated at a distance of 5 $\overset{˚}{A}$ between the two proteins based on the five-five rule. The areas of the complexes were 268∼610 ${\overset{˚}{A}}^{2}$. The binding surface areas were also obtained using ePISA, which calculates the area based on the solvation and represents the three-dimensional contact area. Thus, the sectional area is the two-dimensional projection plan of the three-dimensional actual binding surface. Therefore, if the shape of the binding region is flat, the sectional area will be similar to the three-dimensional solvation area from ePISA. Alternatively, when one protein has a convex surface for binding and the partner protein has a concave surface, the ratio of the ePISA value to the sectional area will be greater than one, whereas if the convexity of the binding surface is similar to a sphere, the ratio will be 4 ($4\pi r^{2}2\pi r^{2}$). This ratio was calculated for the 32 complexes and ranged from 1.9 to 4.3 with an average of 2.8. A value lower suggested a relatively flat binding surface of the proteins in the complex.

The sectional area can also be divided according to the distance between the surfaces of two proteins. Different distances suggest different binding characteristics. In the case of hydrogen bonding, the distance between the surfaces of an atom from each protein has a negative value as the two atoms share a pair of electrons. Meanwhile, if there is a van der Waals interaction with close contact, the distance will be less than 1 $\overset{˚}{A}$. Plus, an interaction mediated by a water molecule should have a space for the water molecule between the two proteins. If there is space for more than two water molecules, this suggests that each protein possesses its own water shell and is not seemingly important for protein interaction. A series of sectional areas can be calculated based on different distance criteria. For example, three sectional area values from the 32 protein complexes were generated using the distance criteria of 1, 3, or 5 $\overset{˚}{A}$ (Table 2). As expected, longer distances produced a larger area like a series of contour lines. By subtracting the area of a shorter distance from the area of a longer distance, the total sectional area can be divided into a series of areas according to the distance, such as ∼1 $\overset{˚}{A}$, 1∼3 $\overset{˚}{A}$, and 3∼5 $\overset{˚}{A}$ (Table 2). The distance less than 1 $\overset{˚}{A}$ implies the tight interaction between two proteins with hydrogen bond or van der Waals contact. In the analyzed 32 protein–protein interactions, average 24% of binding area was less than 1 $\overset{˚}{A}$ compared to the area of 5 $\overset{˚}{A}$ from the five-five rule, and rest of the binding area would have certain flexibility (Table 2).

## 6. Conclusions

The contact region between two protein molecules in a protein complex is usually complementary in shape for each molecule. If one protein has prominence in the region, there will be a depression in the counter part of the other protein. Thus, the flatness or ruggedness of the contact region would be similar for those two protein molecules. Although computing the area of the contact region is useful for estimating the binding strength between the molecules, it is not effective for presenting the ruggedness of the region. A flattened base area of the contact region was necessary to evaluate the three-dimensional ruggedness by comparing with the contact area. A criterion for protein binding direction was required to define the flattened area. Therefore, this paper presented a method to compute the binding direction between two molecules and then measure the two-dimensional contact area based on the binding direction. A novel concept of the sectional contact area between two protein molecules in a protein complex was also introduced, which is useful for evaluating the properties of the protein–protein interaction, such as the degree of prominence and depression on the binding surface, and determining the area depending on the characteristics of the interaction.

## Figures and Tables

**Table 1:** Computed binding directions and area ratios.

PDB Id.	Binding Direction			Sectional Area (${\overset{˚}{A}}^{2}$) 5 $\overset{˚}{A}$ Cutoff Distances	Surface Area from PISA (${\overset{˚}{A}}^{2}$)	Area Ratio (Surface/Sectional)
	x	y	z			
1A19	−0.858840	−0.479551	−0.180066	269.4	501.9	1.9
1A52	−0.294080	-0.000000	-0.955781	607.5	1534.4	2.5
1AY7	0.463474	0.674404	−0.574779	272	616.9	2.3
1BUC	0.330109	0.130558	0.934870	473.2	1884.5	4
1DEH	0.280917	−0.486684	−0.827179	461.4	1,647.3	3.6
1GLP	0.577662	0.641967	0.504168	543.6	1279.7	2.4
1GLQ	0.582690	0.700540	0.411966	526.9	1281.1	2.4
1GSY	0.818358	0.545212	−0.181752	515.2	1191.1	2.3
1HDX	0.434733	−0.480141	−0.761887	439	1577.3	3.6
1HDY	0.350059	−0.606471	−0.713899	469	1680.9	3.6
1HSZ	−0.440008	0.553178	0.707380	496	1671.5	3.4
1HTB	0.370719	−0.642265	−0.670867	477.4	1655.9	3.5
1U3V	−0.369964	0.502553	0.781388	473.5	1612.7	3.4
2BJ4	−0.412982	−0.097308	−0.905526	482.1	1506.6	3.1
2GLR	0.603841	0.564992	0.562281	548.7	1279.1	2.3
2HXX	0.4322447	0.788672	0.437020	268.3	541.9	2
2I0J	−0.976684	−0.195474	−0.088755	569.9	1534.6	2.7
2JFA	−0.162518	−0.939318	0.302110	540.3	1536.5	2.8
2JIF	0.234259	0.932876	0.273614	541.8	1641.9	3
2OA7	0.697742	−0.669865	−0.253843	545.9	1247.5	2.3
2POG	0.537877	−0.842968	−0.009662	610.3	1470.9	2.4
2Q70	0.557888	−0.827531	−0.062875	599.7	1482.1	2.5
2UZ8	−0.735231	−0.674951	−0.062266	298.3	1143.6	3.8
3M8N	0.908404	0.416203	0.039722	537.5	1414.5	2.6
3MPJ	0.958105	−0.034863	0.284287	427.8	1860.5	4.3
3O76	0.721400	0.533321	0.441758	552.3	1226.5	2.2
4BL7	0.173459	−0.233176	−0.956839	514.5	1261.4	2.5
4BVX	−0.169235	0.268273	−0.948362	473.5	1224.5	2.6
4BVY	0.447971	0.227383	0.864650	468.6	1211.9	2.6
4L1F	0.637533	−0.323275	0.699318	550.1	1667.9	3
4M9A	−0.351462	0.830645	−0.431860	575.2	1632.1	2.8
4N5F	0.681050	0.648964	0.339142	553.7	1325.9	2.4
Mean				490.1	1385.8	2.8

**Table 2:** The sectional contact area in protein–protein complexes.

PDB Id.	Sectional Area (${\overset{˚}{A}}^{2}$) Cutoff Distances			Partial Area(${\overset{˚}{A}}^{2}$) for a Section in Distance of			Percentage of the Area in Distance of		
	1 $\overset{˚}{A}$	3 $\overset{˚}{A}$	5 $\overset{˚}{A}$	∼1 $\overset{˚}{A}$	1∼3 $\overset{˚}{A}$	3∼5 $\overset{˚}{A}$	∼1 $\overset{˚}{A}$	1∼3 $\overset{˚}{A}$	3∼5 $\overset{˚}{A}$
1A19	44.1	179.2	269.4	44.1	135.2	90.1	16.4	50.2	33.4
1A52	109.9	371.8	607.5	109.9	261.9	235.7	18.1	43.1	38.8
1AY7	68.7	187.1	272	68.7	118.4	84.9	25.3	43.5	31.2
1BUC	111.2	366.8	473.2	111.2	255.5	106.4	23.5	54.0	22.5
1DEH	88.8	302.3	461.4	88.8	213.6	159.1	19.2	46.3	34.5
1GLP	139.6	398.8	543.6	139.6	259.2	144.8	25.7	47.7	26.6
1GLQ	135	391.3	526.9	135	256.3	135.6	25.6	48.6	25.7
1GSY	139.2	364.5	515.2	139.2	225.4	150.7	27.0	43.7	29.2
1HDX	100.4	308.3	439	100.4	208	130.7	22.9	47.4	29.8
1HDY	114.7	331.2	469	114.7	216.5	137.8	24.5	46.2	29.4
1HSZ	125.1	343.8	496	125.1	218.8	152.2	25.2	44.1	30.7
1HTB	119.1	335.8	477.4	119.1	216.8	141.6	24.9	45.4	29.7
1U3V	121.5	327.6	473.5	121.5	206	145.9	25.7	43.5	30.8
2BJ4	157.5	323.3	482.1	157.5	165.8	158.8	32.7	34.4	32.9
2GLR	138.9	400.1	548.7	138.9	261.2	148.5	25.3	47.6	27.1
2HXX	61.7	171.8	268.3	61.7	110.2	96.5	23.0	41.1	36.0
2I0J	134.4	361.7	569.9	134.4	227.3	208.2	23.6	39.9	36.5
2JFA	113.8	325.2	540.3	113.8	211.4	215.1	21.1	39.1	39.8
2JIF	130.3	393.4	541.8	130.3	263.1	148.4	24.0	48.6	27.4
2OA7	136.6	400.7	545.9	136.6	264.1	145.3	25.0	48.4	26.6
2POG	130	362.4	610.3	130	232.5	247.9	21.3	38.1	40.6
2Q70	132.5	355.7	599.7	132.5	223.3	244	22.1	37.2	40.7
2UZ8	75.3	208.3	298.3	75.3	132.9	90.1	25.2	44.6	30.2
3M8N	141.2	341.7	537.5	141.2	200.5	195.8	26.3	37.3	36.4
3MPJ	107.4	318.7	427.8	107.4	211.3	109.1	25.1	49.4	25.5
3O76	149	402.7	552.3	149	253.7	149.6	27.0	45.9	27.1
4BL7	128.2	334.7	514.5	128.2	206.5	179.8	24.9	40.1	34.9
4BVX	122.9	307.8	473.5	122.9	184.9	165.7	26.0	39.0	35.0
4BVY	121.7	311.4	468.6	121.7	189.8	157.2	26.0	40.5	33.5
4L1F	151.1	396.5	550.1	151.1	245.4	153.6	27.5	44.6	27.9
4M9A	143.6	387.7	575.2	143.6	244.1	187.6	25.0	42.4	32.6
4N5F	105.6	365.1	553.7	105.6	259.4	188.7	19.1	46.8	34.1
Average	118.7	333.7	490.1	118.7	215	156.4	24.2	44.0	31.8
